# Probiotics mixture reinforces barrier function to ameliorate necrotizing enterocolitis by regulating PXR-JNK pathway

**DOI:** 10.1186/s13578-021-00530-7

**Published:** 2021-01-19

**Authors:** Xiuhao Zhao, Jin Zhou, Wenhua Liang, Qingfeng Sheng, Li Lu, Tong Chen, Jianglong Chen, Kezhe Tan, Zhibao Lv

**Affiliations:** 1 Department of General Surgery, Shanghai Children’s Hospital, Shanghai Jiao Tong University, 355 Luding Road, Putuo, Shanghai, China; 2 Shanghai Institute of Immunology, Shanghai Jiao Tong University School of Medicine, Shanghai, China

**Keywords:** Necrotizing enterocolitis, Intestinal barrier, Probiotic, Pregnane X receptor

## Abstract

**Background:**

Intestinal dysbiosis is believed to be one of the factors inducing neonatal necrotizing enterocolitis (NEC). Probiotics have been employed to treat NEC in a number of animal experiments and clinical trials, and some significant benefits of utilizing probiotics for the prevention or alleviation of NEC have been confirmed. However, the mechanism underlying the efficacy of probiotics in treating NEC has not been elucidated.

**Results:**

Impairment of the intestinal barrier, which was characterized by the decreased expression of tight junction components, was observed in the pathogenesis of NEC. The probiotic mixture alleviated this intestinal damage by enhancing the function of the barrier. Meanwhile, the probiotics remodeled the composition of the intestinal microbiota in NEC mice. Furthermore, increased expression of the pregnane X receptor (PXR) was observed after treatment with the probiotic mixture, and PXR overexpression in Caco-2 cells protected the barrier from lipopolysaccharide (LPS) damage. Further research showed that PXR could inhibit the phosphorylation of c-Jun N-terminal kinase (JNK) and could increase the expression of tight junction components.

**Conclusions:**

Our study confirmed that probiotics could ameliorate intestinal lesions by enhancing the function of the mucosal barrier. Specifically, probiotics may target PXR, which may subsequently enhance the expression of tight junction components by inhibiting the phosphorylation of JNK and enhance the function of the barrier.

## Background

Necrotizing enterocolitis (NEC) is a severe disease that occurs in neonatal preterm infants and has potentially life-threatening consequences. The mean prevalence of NEC is approximately 7% among infants weighing < 1500 g, and the estimated mortality rate of NEC ranges from 20 to 30% [1]. The possible risk factors leading to NEC include immaturity of the intestine and immune system, intestinal ischemia, formula feeding, and intestinal dysbacteria. These factors might result in disintegration of the intestinal epithelium and dysfunction of the immature mucosal barrier, which eventually leads to rampant bacterial translocation into systemic circulation and the occurrence of sepsis [2, 3]. Therefore, methods to reinforce the epithelial barrier could be employed for the prevention of NEC.

Intestinal barrier function is mediated by the apical junction complex (AJC), which is composed of tight junctions (TJs), adherent junctions (AJs), and desmosomes. Tight junctions are the primary determinant of paracellular permeability and are composed of the claudin family, occludin, and zonula occludens (ZO) family [4]. A number of studies have reported that changes in tight junctions, which feature a reduction in occludin and an increase in claudin-3, are responsible for the development of NEC [5, 6]. Hence, strategies for regulating tight junctions are believed to be effective methods for preventing neonatal necrotizing enterocolitis.

Several studies have shown a link between dysbacteria in preterm infants and the development of NEC [7, 8]. For example, some reports described a decreased relative abundance of *Firmicutes* and an increased relative abundance of *Enterobacteriaceae* in NEC patients [8–10]. Therefore, various probiotics have been tested for the ability to regulate intestinal flora balance to prevent NEC [11]. Although randomized controlled trials (RCTs) with different probiotics have shown contradictory results, several meta-analyses have consistently found a significant benefit of probiotics for the prevention of NEC and related mortality [12–14]. Meanwhile, animal experiments have suggested various mechanisms by which probiotics protect the immature intestine from inflammation and impairment, including competition with pathogenic bacteria, regulation of cellular immunity, and downregulation of proinflammatory cytokines [15]. Notably, the promotion of barrier maturation and function has been considered an important mediator of probiotic function during NEC pathogenesis [16]. Therefore, in this study, we applied a commensal probiotic mixture during NEC pathogenesis and investigated the underlying mechanism by which probiotics regulate the intestinal epithelial barrier.

Orphan nuclear receptors are known as key intermediates for signaling from microorganisms to intestinal epithelial cells (IECs), which depend on bacteria themselves, bacterial components, or bacterial metabolites [17]. In this study, we focused on the pregnane X receptor (PXR, gene named *Nr1i2*), a nuclear receptor and xenobiotic sensor that is predominantly expressed in the liver and intestine [18]. Our results showed that probiotic treatment could upregulate the expression of PXR and that overexpression of PXR could elevate the expression of tight junction components and enhance intestinal epithelial barrier functions through the inhibition of c-Jun N-terminal kinase (JNK) phosphorylation. These results help to elucidate how probiotics alleviate NEC damage by enhancing the functions of the intestinal barrier.

## Results

### Tight junctions were altered during NEC pathogenesis

**Fig. 1:**
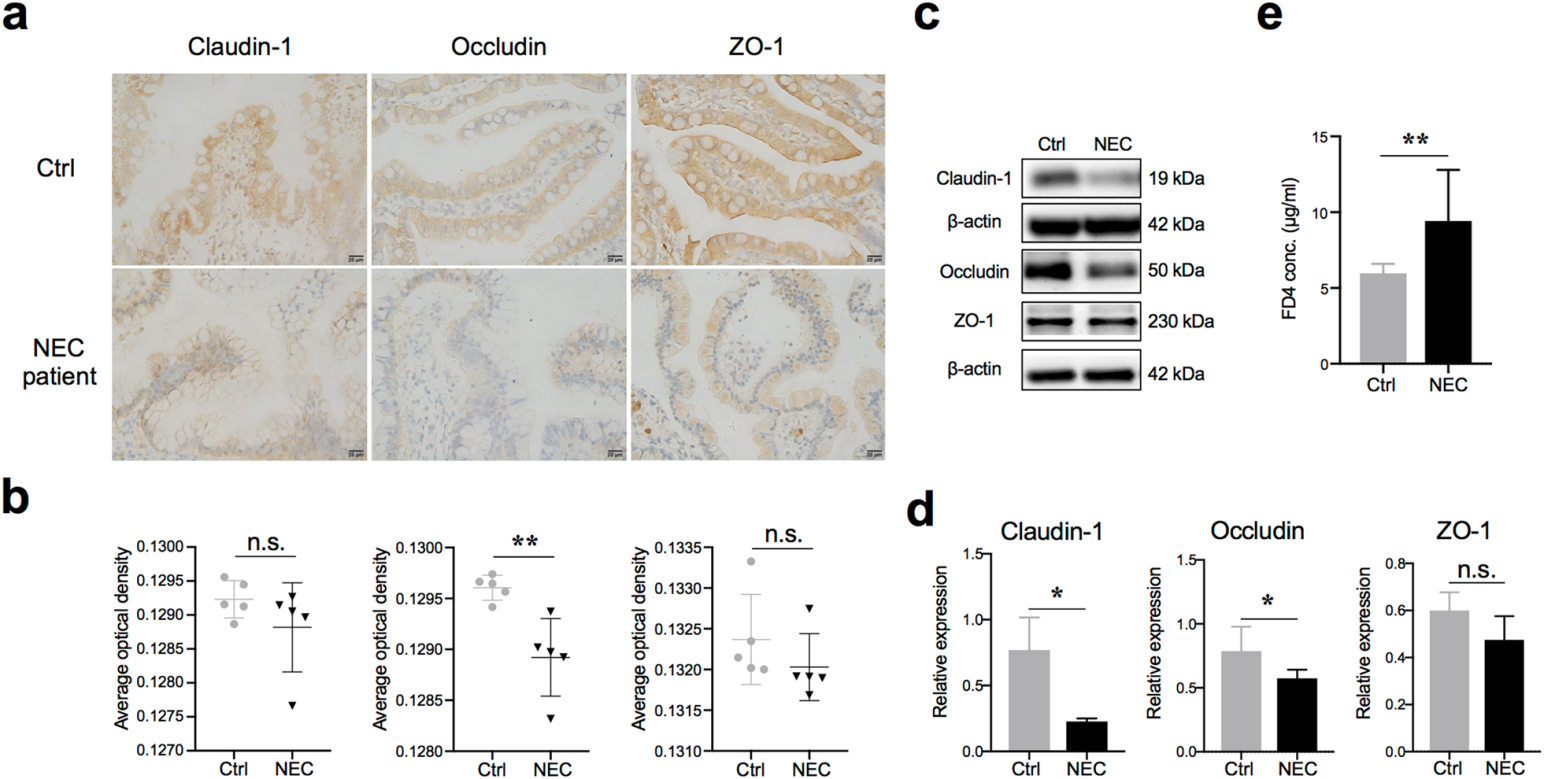
Intestinal barrier was destroyed in NEC patients and mice. **a** Representative sections from ileac tissue of Ctrl or NEC patients stained with anti-claudin-1, anti-occludin, and anti-ZO-1 antibodies. Neonatal intestinal atresia patients were used as controls. **b** Statistical analysis of the average optical density of immunohistochemical staining images of (**a**) (n = 5 per group). **c** and **d** Western blot analysis of claudin-1, occludin, and ZO-1 protein levels in small intestinal epithelial cells of NEC mice and relative gray calculation (n = 5 ~ 6 per group, representative data from four independent experiments). **e** The concentration of FITC-dextran 4 kDa (FD4) in the serum of Ctrl or NEC mice. Ctrl, dam-fed, n = 5 ~ 6 per group. Four independent experiments were performed. Data are shown as mean values ± SD. Statistical analyses were performed with Student’s two-tailed unpaired *t*-test. *p < 0.05; **p < 0.01; n.s., not significant. Scale bars represent 20 μm in (**a**)

To determine whether the intestinal epithelial barrier of NEC patients is altered, we first employed immunohistochemistry (IHC) to investigate the expression of tight junction components (claudin-1, occludin, and ZO-1) in patient tissues. Compared with the control group, which consisted of patients with congenital intestinal atresia, the NEC patients showed significantly decreased expression of occludin (Fig. 1a, b). Similar results were also observed in the neonatal mouse model of NEC. The expression of both claudin-1 and occludin significantly decreased in the small intestinal epithelial cells of NEC mice (Fig. 1c, d). Consistent with this result, a significant increase in serum FITC-dextran 4 kDa (FD4), which is a marker of gut permeability in vivo, was observed in NEC mice (Fig. 1e). Taken together, these results suggested that the intestinal barrier was impaired during NEC pathogenesis.

### Probiotics lessened the severity of NEC and enhanced intestinal barrier function

**Fig. 2:**
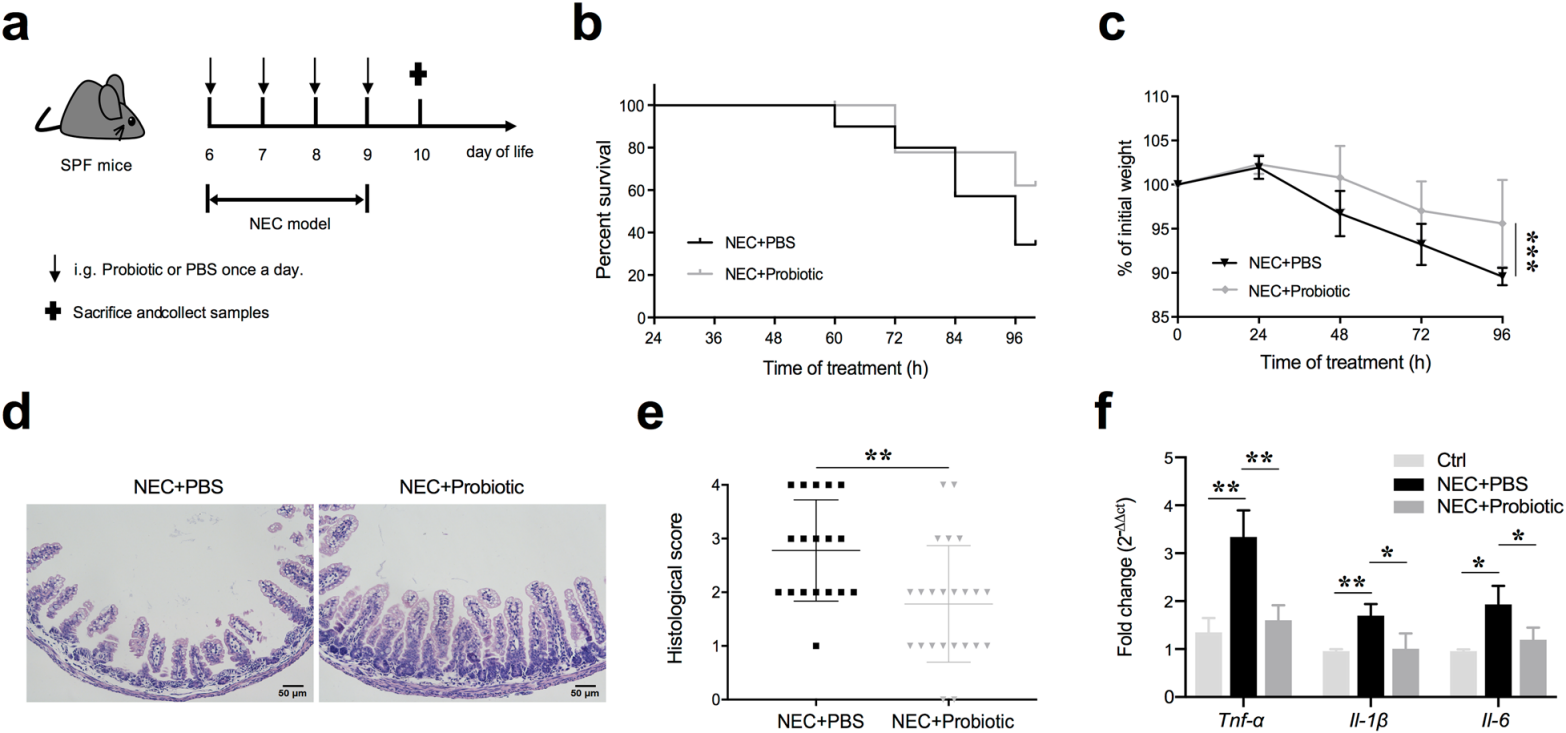
Probiotics ameliorated NEC injury. **a** Diagram of the experimental design performed in SPF 6 ~ 7-day-old C57BL/6 mice. **b** Survival curve of newborn mice with NEC (n = 9 ~ 10 per group, representative data from three independent experiments). **c** Percentage of initial weight (n = 5 ~ 6 per group, representative data from three independent experiments). **d** Representative hematoxylin and eosin staining sections of the terminal ileum from PBS- or probiotic-treated mice with NEC. **e** Histological score measuring the severity of tissue lesions in the terminal ileum (data were pooled from three independent experiments). **f** Real-time qPCR demonstrating the expression of pro-inflammatory genes in the terminal ileum (Ctrl, dam-fed mice; data shown as fold change relative to Ctrl, n = 5 ~ 6 per group, representative data from three independent experiments). Data are expressed as mean values ± SD. Statistical analyses were performed with Student’s two-tailed unpaired *t*-test in (**e**) or one-way ANOVA in (**f**) or two-way ANOVA in (**c**). *p < 0.05; **p < 0.01; ***p < 0.001. Scale bars represent 50 μm in (**d**)

Since changes in the intestinal microbial community have already been observed in NEC patients and probiotics may relieve NEC injury by regulating intestinal flora [8], we investigated whether the probiotic mixture used in this study could alleviate intestinal lesions in NEC mice. This probiotic mixture has been recommended in the clinic for the treatment of children with intestinal dysfunction but not for NEC patients. As the results showed, oral gavage of probiotics was found to effectively prevent weight loss in NEC mice, although the survival rate was not improved significantly (Fig. 2a–c). Hematoxylin and eosin staining of the terminal ileum, as well as histopathological scores, demonstrated a significant reduction in histological injury in probiotic-treated NEC mice (Fig. 2d, e). Furthermore, the probiotic mixture also significantly suppressed the expression of *Tnf-α*, *Il-1β*, and *Il-6* in terminal ileac tissues, which were elevated in NEC patients [19] (Fig. 2f). These results indicated that the probiotic mixture could ameliorate intestinal damage during NEC.

**Fig. 3:**
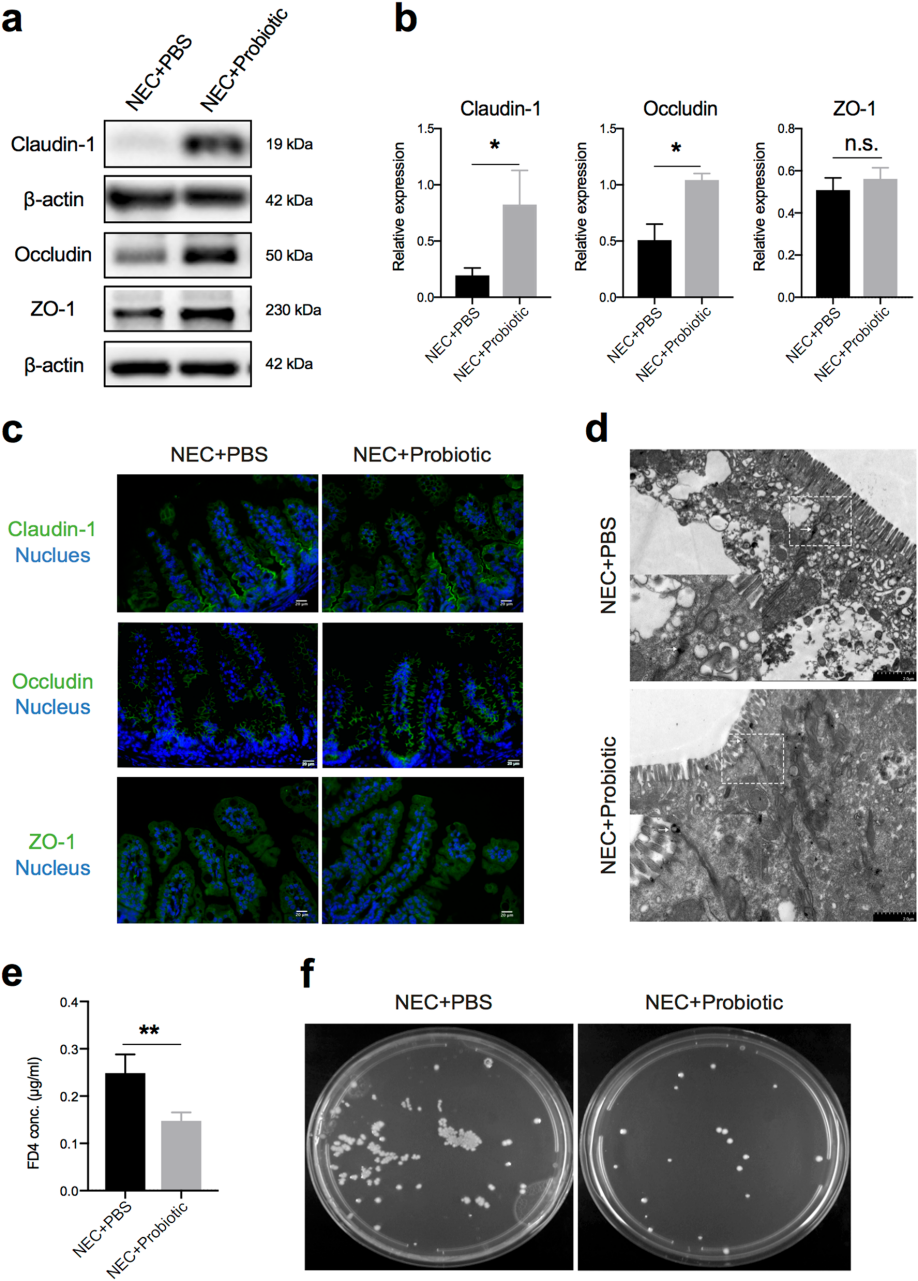
Probiotics alleviated gut barrier permeability and bacterial translocation. **a** Representative images of claudin-1, occludin, and ZO-1 expression in small intestinal epithelial cells from three independent experiments. **b** Quantification of claudin-1, occludin, and ZO-1 expression (n = 5 ~ 6 per group). **c** Representative immunofluorescence images of claudin-1, occludin, and ZO-1 staining in the terminal ileum. **d** Representative TEM images of ileum enterocytes revealing cell–cell junctional complexes. The inset shows magnified views of the tight junction. **e** Serum concentrations of FD4 in NEC mice treated with PBS or probiotics (n = 5 ~ 6 per group, representative data from three independent experiments). **f** Representative agar plates from NEC + PBS and NEC + probiotic splenic lysates, plated and incubated for 24 h. Data are expressed as mean values ± SD. Statistical analyses were performed with Student’s two-tailed unpaired *t*-test in (**b**, **e**), *p < 0.05; **p < 0.01; n.s., not significant; FD4, FITC-dextran 4 kDa. Scale bars represent 2 μm in (**d**)

As the probiotic mixture could protect the intestine from NEC damage, we further examined whether the intestinal barrier and its functions were also restored. Two components of tight junctions, claudin-1 and occludin, which were reduced in NEC mice, were preserved in probiotic-treated mice (Fig. 3a, b). Consistent with this finding, similar results were confirmed by indirect immunofluorescence in the terminal ileum (Fig. 3c). We also employed transmission electron microscopy (TEM) to examine the ultrastructure of the cell–cell junctional complex in the terminal ileum. The images showed that the intercellular space in probiotic-treated mice exhibited less penetration of the electrodense dye ruthenium red than that in PBS-treated mice, suggesting a more complete structure of tight junctions (Fig. 3d, white arrows). To further confirm the improvement of barrier functions, gut permeability in vivo was assessed by measuring serum FD4 concentrations. Unsurprisingly, compared with PBS-treated mice, FD4 levels were reduced significantly in probiotic-treated mice, indicating that intestinal permeability was improved (Fig. 3e). Meanwhile, agar plates incubated with splenic lysates from NEC mice showed lower numbers of colony-forming units for probiotic-treated mice, further confirming that the probiotic mixture decreased gut permeability and prevented bacterial translocation to extraintestinal organs (Fig. 3f). Taken together, these results suggested that the probiotic mixture could enhance tight junction functions and prevent bacterial translocation.

### Probiotics remodeled the microbiota composition of the small intestine

**Fig. 4:**
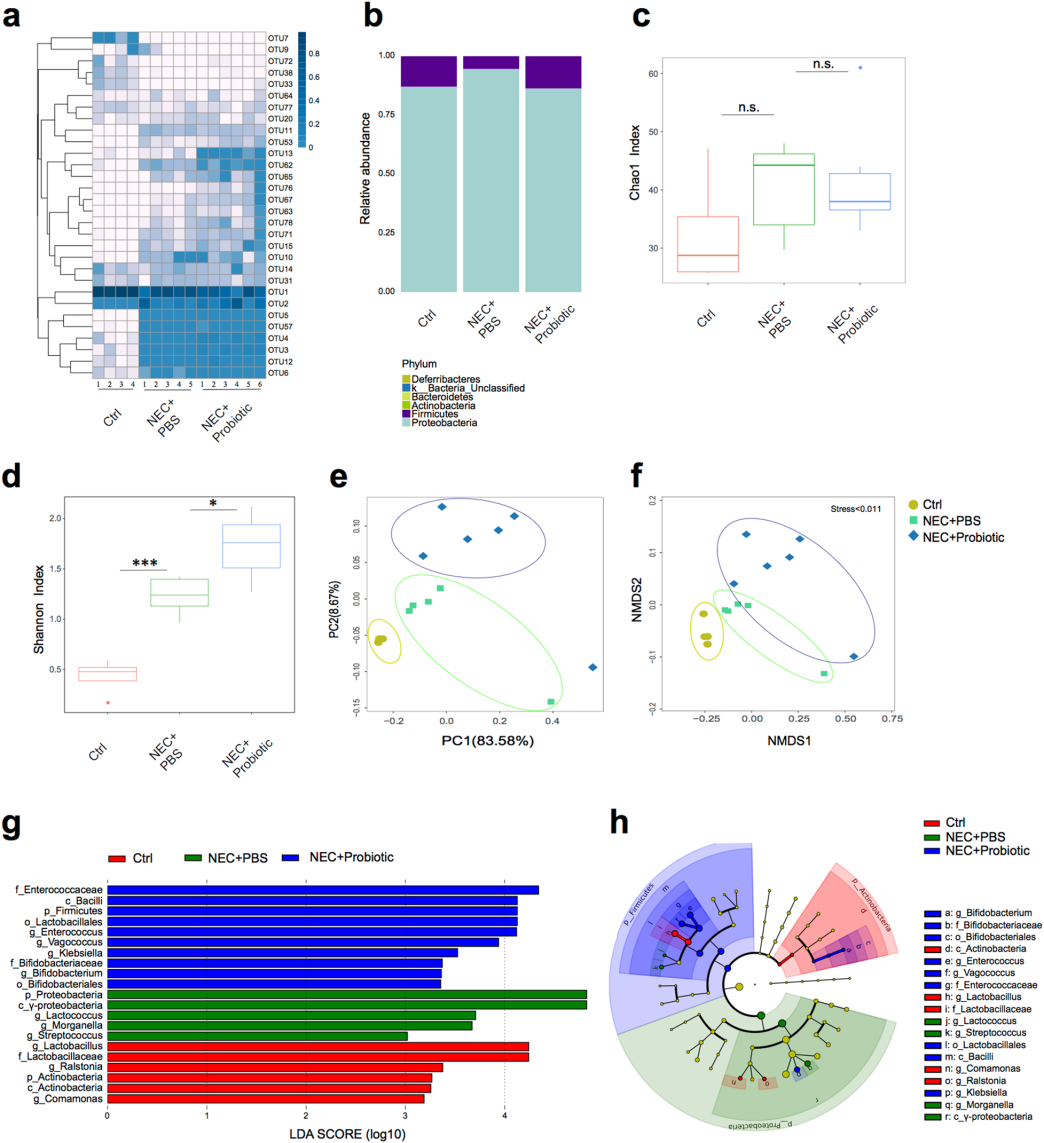
Probiotics regulated microbial community structure. **a** Uncluster heatmap of the top 30 OTUs. **b** Average abundance of the microbiota at the phylum level. **c** and **d** Alpha diversity, as represented by the Chao1 index and Shannon index. Boxes represent the interquartile range (IQR), with the inside line representing the median. **e** Effect of probiotic treatment on microbiota composition in principal coordinates analysis (Bray–Curtis dissimilarities). **f** Nonmetric multidimensional scaling (NMDS) based on the distance between the matrix Brary-Curtis. **g** and **h** Taxonomic biomarkers of three groups were identified by LDA scores using LEfSe and presented as a cladogram. Color signifies the group in which a differentially abundant taxon is enriched. Statistical analyses were performed with Student’s two-tailed unpaired *t*-test in (**c**) and (**d**). *p < 0.05; ***p < 0.001; n.s., not significant

In addition to enhancing intestinal barrier functions, the probiotic mixture might also remodel the microbiota composition of the gut, which might be directly or indirectly beneficial for the intestinal barrier [20, 21]. To confirm this possibility, the composition and diversity of the microbiota in the small intestine were analyzed by 16S rRNA sequencing. As shown by the unclustering and abundance of the 30 most abundant operational taxonomic units (OTUs), the patterns of the NEC + PBS and NEC + probiotic groups were different from those of the control (Fig. 4a). To further characterize the bacterial community, we assessed the relative abundance at the phylum level. We found that the NEC + probiotic and control groups showed a similar relative abundance of *Proteobacteria* and *Firmicutes* which, on average, accounted for nearly 100% of all reads (Fig. 4b). The NEC + PBS group contained fewer *Firmicutes* and more *Proteobacteria*, which is consistent with the phenomena observed in NEC patients [8]. Further analysis of bacterial diversity by the Shannon index revealed the highest bacterial diversity in the NEC + probiotic group compared with the other groups, although no significant difference was observed among the three groups by calculation with the Chao 1 index (Fig. 4c, d). Both principal coordinate analysis (PCoA) and nonmetric multidimensional scaling (NMDS) demonstrated an apparent separation of the three groups, suggesting that they had different compositions of intestinal microbiota (Fig. 4e, f). The analysis of LEfSe (linear discriminant analysis effect size), which is employed to identify the key bacterium affecting the discrimination between groups, identified 21 discriminatory taxa as key discriminants with an LDA (linear discriminant analysis) score higher than 3.0 (Fig. 4g, h). The phyla *Proteobacteria* and class *Gammaproteobacteria* showed especially high LDA scores in the NEC + PBS group. Importantly, in the probiotic-treated group, the differentially abundant strains primarily included the genus *Bifidobacterium*, family *Bifidobacteriaceae*, order *Lactobacillales*, phyla *Firmicutes*, genus *Enterococcus*, and class *Bacilli* (Fig. 4g, h), and most of them were the components of the probiotic mixture that we used. Taken together, these results suggested that the probiotics colonized steadily in the small intestine of newborn mice and remodeled the microbiota community in NEC mice.

### Probiotics induced PXR activation

**Fig. 5:**
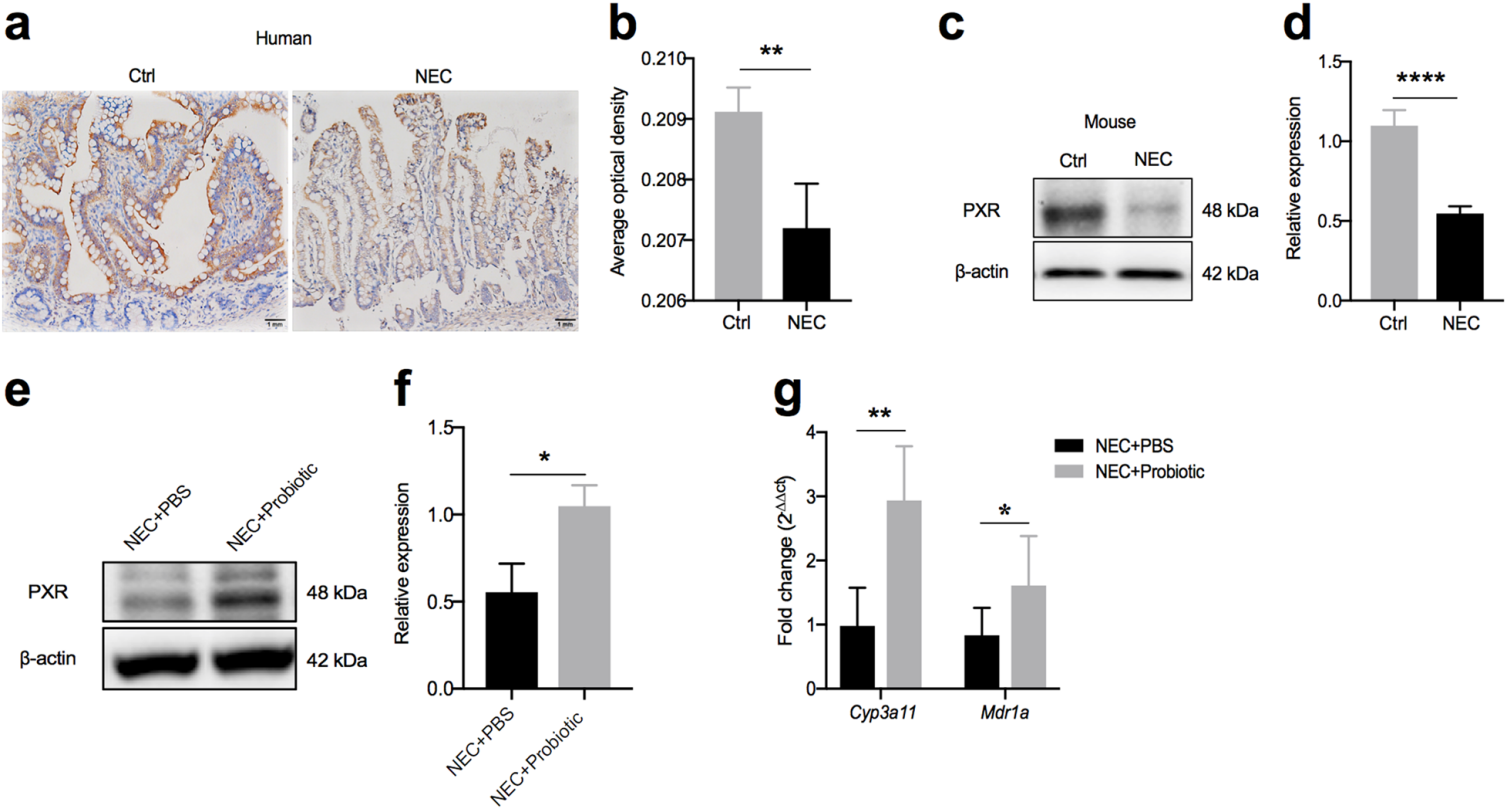
Probiotics enhanced the expression of PXR.** a** and **b** Representative sections from ileac tissue of Ctrl or NEC patients stained with anti-PXR antibody and statistical results of the average optical density of staining images (Ctrl, neonatal intestinal atresia patient; n = 5 per group). **c** and **d** Western blot analysis of PXR protein levels in epithelial cells of the small intestine and their relative gray calculations (n = 5 ~ 6 per group, three independent experiments). **e** and **f** Western blot analysis of PXR protein levels in NEC mice treated with PBS or probiotics and their relative gray calculations (n = 5 ~ 6 per group, representative data from three independent experiments). **g**, Real-time qPCR analysis of PXR target gene (*Cyp3a11* and *Mdr1a*) mRNA expressions in the terminal ileum (n = 5 per group). All graphs show mean values ± SD. Statistical analyses were performed with Student’s two-tailed unpaired *t*-test in (**b**, **d, f, g**), *p < 0.05; **p < 0.01; ****p < 0.0001; n.s., not significant. Scale bars represent 1 mm in (**a**)

Since PXR has been demonstrated to play an important role in protecting the barrier in intestinal or liver disease [16, 22], we investigated whether PXR also plays a role in NEC pathogenesis. As shown in Fig. 5a and b, the expression of PXR was decreased significantly in NEC patients. Meanwhile, a reduction in PXR expression was also observed in the intestinal epithelial cells of NEC mice (Fig. 5c, d). Importantly, when mice were treated with a probiotic mixture during NEC modeling, the expression of PXR was significantly enhanced (Fig. 5e, f), and the transcription levels of PXR target genes, such as *Cyp3a11* and *Mdr1a*, were also increased (Fig. 5g). These results indicated that the probiotic mixture might be an activator of PXR, which might help to relieve the pathological injury related to NEC.

### Activation of PXR improved barrier function in Caco-2 cells

**Fig. 6:**
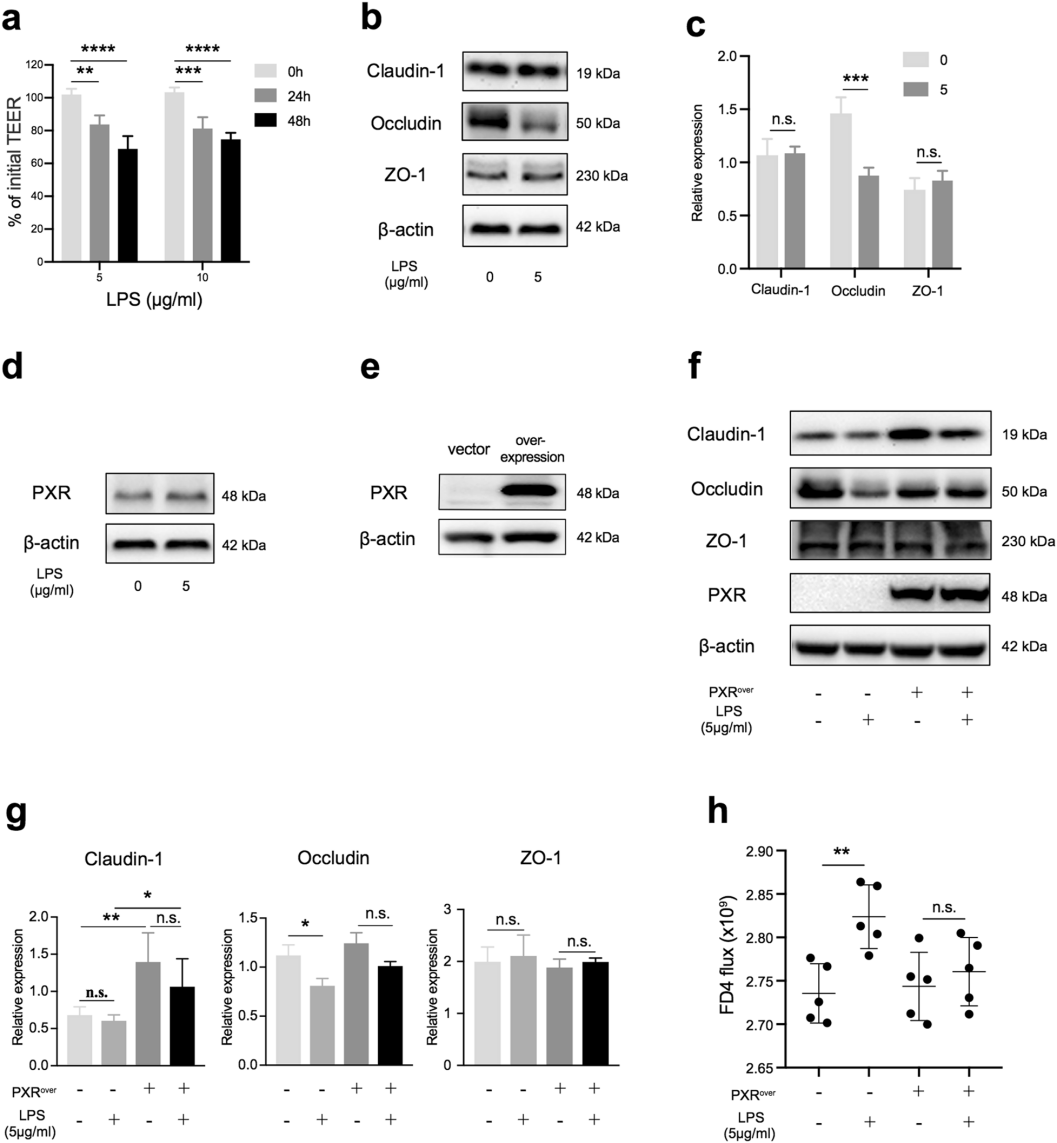
PXR protected barrier function from LPS damage. **a** TEER changes in Caco-2 cell monolayers treated with LPS for 24 or 48 h. **b** and **c** Western blot analysis of claudin-1, occludin, and ZO-1 protein levels in Caco-2 cells treated with LPS (n = 4 per group, representative data from three independent experiments). **d** Western blot analysis of PXR in Caco-2 treated with LPS. **e** Confirmation of PXR overexpression by Western blot. **f** and **g** Western blot analysis of the components of tight junctions (Claudin-1, Occludin, and ZO-1) in Caco-2 cells treated with LPS for 48 h (n = 4 per group, representative data from three independent experiments). **h** FD4 flux of basolateral medium was measured after Caco-2 cells were treated with LPS or not treated for 48 h (n = 5 per group, representative data from four independent experiments). Data are expressed as mean values ± SD. Statistical analyses were performed with Student’s two-tailed unpaired *t*-test in (**c** and **g**), *p < 0.05; **p < 0.01; n.s., not significant; TEER, transepithelial electrical resistance; FD4, FITC-dextran 4 kDa

To further confirm the role of PXR in enhancing barrier function and reveal its mechanism, we investigated it in Caco-2, a human-derived intestinal epithelial cell line that can be used to simulate the barrier in vitro [23]. To model this function, Caco-2 cells were seeded in Transwell inserts and cultured to form monolayer cells for the permeability assay. The results showed that transepithelial electrical resistance (TEER) decreased significantly when monolayer cells were treated with LPS at 5 or 10 μg/ml for 48 h (Fig. 6a), suggesting damage to the monolayer by LPS. Meanwhile, we measured changes in tight junction proteins. The results of this experiment showed that the expression of occludin was markedly depressed by LPS treatment at 5 μg/ml for 48 h (Fig. 6b, c). Meanwhile, we found that LPS did not have an effect on the PXR expression (Fig. 6d). To further investigate the role of PXR in regulation of the intestinal barrier, we established a stable PXR overexpression cell line (Fig. 6e). Interestingly, when Caco-2 cells overexpressed PXR, claudin-1 expression was elevated significantly, both in the absence and presence of LPS. Furthermore, PXR overexpression attenuated the reduction in occludin induced by LPS (Fig. 6f, g). Consistent with this finding, FD4 flux, an indicator of epithelial paracellular permeability to uncharged macromolecules, did not increase when the PXR-overexpressing Caco-2 monolayers were treated with LPS (Fig. 6h). Taken together, these results suggested that PXR could enhance barrier function by regulating the tight junction complex.

### PXR could inhibit the phosphorylation of JNK

**Fig. 7:**
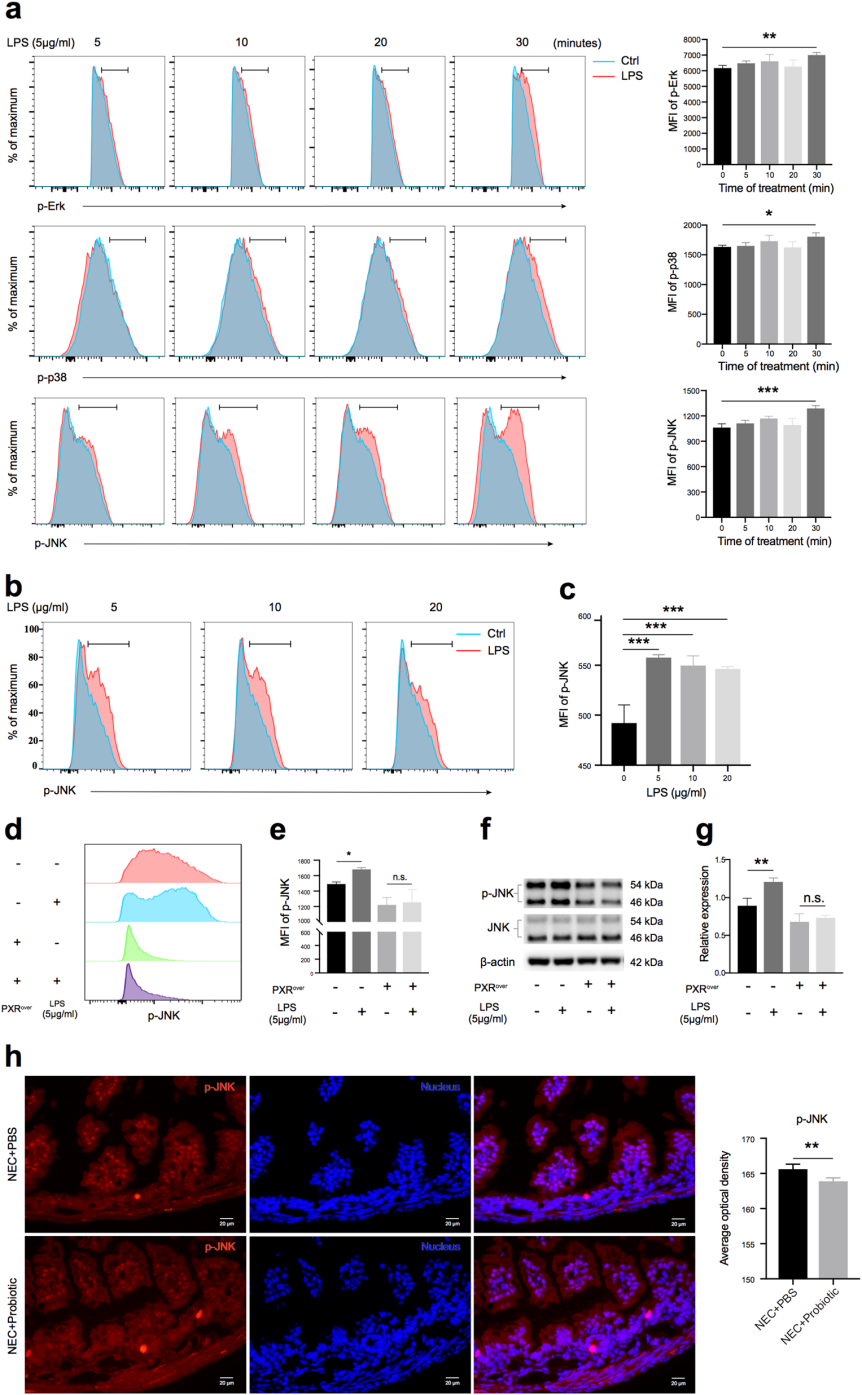
PXR inhibited the phosphorylation of JNK. **a** Phosphorylation of Erk, p38, and JNK detected by flow cytometry in Caco-2 cells treated with LPS for different times and statistical analysis of MFI. **b** and **c** Phosphorylation of JNK in Caco-2 cells treated with different concentrations of LPS for 30 min and statistical analysis of MFI. **d** and **e** Phosphorylation of JNK in empty or PXR-overexpressing Caco-2 cells treated with LPS and statistical analysis of MFI. **f** and **g** Phosphorylation of JNK in Caco-2 cells was detected by Western blotting and statistical analysis of relative expression. **h** Phospho-JNK in the terminal ileum of mice was detected by immunofluorescence and statistical analysis of average optical density. Data are expressed as mean values ± SD. n = 3 ~ 5 per group, representative data from three independent experiments. Statistical analyses were performed with Student’s two-tailed unpaired *t*-test, *p < 0.05; **p < 0.01; ***p < 0.001; n.s., not significant; JNK, c-Jun N-terminal kinase; MFI, mean fluorescence intensity. Scale bars represent 20 μm in (**h**)

As the MAPK pathway could affect tight junctions and cause dysfunction of the intestinal barrier [22, 24, 25], we further examined whether PXR could regulate MAPK pathway signaling. After Caco-2 cells were treated with LPS at 5 μg/ml for 30 min, we observed increased phosphorylations of Erk and p38 and a notable increase in phospho-JNK level (Fig. 7a–c). Previous study has demonstrated that when PXR was activated by rifaximin, a specific ligand for PXR, the phosphorylation levels of Erk and p38 decreased significantly in Caco-2 cells [26], but whether the phosphorylation of JNK was also affected by PXR was not reported. Here, we demonstrated that when Caco-2 cells overexpressed PXR, LPS treatment could not induce the phosphorylation of JNK, as shown by the results of flow cytometry and Western blot analysis (Fig. 7d–g). Furthermore, we found that the phosphorylation of JNK which localized in nucleus (Fig. 7h and Additional file 1: Fig. S1) was inhibited in the terminal ileum when NEC mice were treated with probiotics (Fig. 7h), which caused PXR to be significantly enhanced (Fig. 5e, f). Taken together, these results indicated that PXR could inhibit the phosphorylation of JNK and thus protect the barrier.

## Discussion

The pathogenesis of NEC has not been elucidated to date, but the immature or defective intestinal epithelial barrier of preterm infants is proposed to be one of the factors that induces NEC [27–29]. Methods for enhancing the intestinal barrier may represent potential strategies for preventing NEC. Recently, the role of commensal bacteria in regulating the intestinal barrier has been emphasized in several reviews [30, 31]. In this study, we applied a probiotic mixture in the pathogenesis process of NEC in a mouse model, and we observed that probiotics could enhance the function of the intestinal barrier and ameliorate the severity of NEC. Furthermore, we found that probiotics could remodel the structure of the intestinal microbiota. Therefore, we proposed that the remodeling of the microbial community by probiotics might help to maintain barrier function and reduce the severity of NEC.

As a xenobiotic sensor and one of the most critical transcription factors, PXR can be activated by steroid, pregnane, bile acids, clinical drugs, dietary supplements, and some environmental pollutants [32–34]. The cellular function of activated PXR is mostly exerted by its direct binding to many genes or crosstalk with other transcriptional factors involved in metabolism modulation, cell cycle arrest, angiogenesis, and inflammation [35]. Recent studies have reported that PXR contributes to regulate the intestinal barrier function [16, 36]. In this study, we observed that the expression of PXR was inhibited during NEC and that probiotics could induce its activity. Meanwhile, we confirmed that PXR could accumulated into the nucleus when Caco-2 cells were treated with rifaximin, an agonist for PXR (Additional file 2: Fig. S2) and overexpression of PXR could increase the expression of the components of tight junctions and enhance barrier functions in vitro. Several studies have demonstrated that activation of PXR increases the expression of growth arrest and DNA damage-inducible 45β (GADD45β), which can inhibit JNKs activation by directly interacting with its upstream kinase MKK7 [37–40]. Here, we revealed that PXR could protect barrier functions by suppressing the phosphorylation of JNK, which might be also associated with increased expression of GADD45β. Taken together, our results indicate that probiotics can ameliorate NEC severity by enhancing intestinal barrier function, which may depend on regulation of the PXR-JNK signaling pathway.

A balance between the intestinal microbiota and host is critical for maintaining homeostasis and preventing anomalous inflammation. In preterm infants, due to the use of formula milk and antibiotics, the vulnerable intestinal community is always exposed to newly colonizing commensal or pathogenic bacteria, which may easily lead to disruption of the bacterial community. Several studies have reported the alteration of gut microbiota with decreased richness and diversity before the onset of NEC [8, 9, 41]. Therefore, we hypothesized that probiotics could remodel the microbial community during NEC pathogenesis, and the results of our study confirmed this hypothesis. In our results, NEC mice showed a high relative abundance of *Proteobacteria* and a low relative abundance of *Firmicutes*, which is also found in NEC patients [10], and the applied probiotics could restore it to a beneficial status. Furthermore, the probiotics increased the bacterial diversity and remodeled the composition of microbiota differently from NEC mice. Although some studies have reported that probiotics are ineffective and not recommended for routine use for NEC [13, 42], the results of our study suggest that probiotic mixture might be useful for regulating the bacterial community and preventing NEC.

## Conclusions

**Fig. 8:**
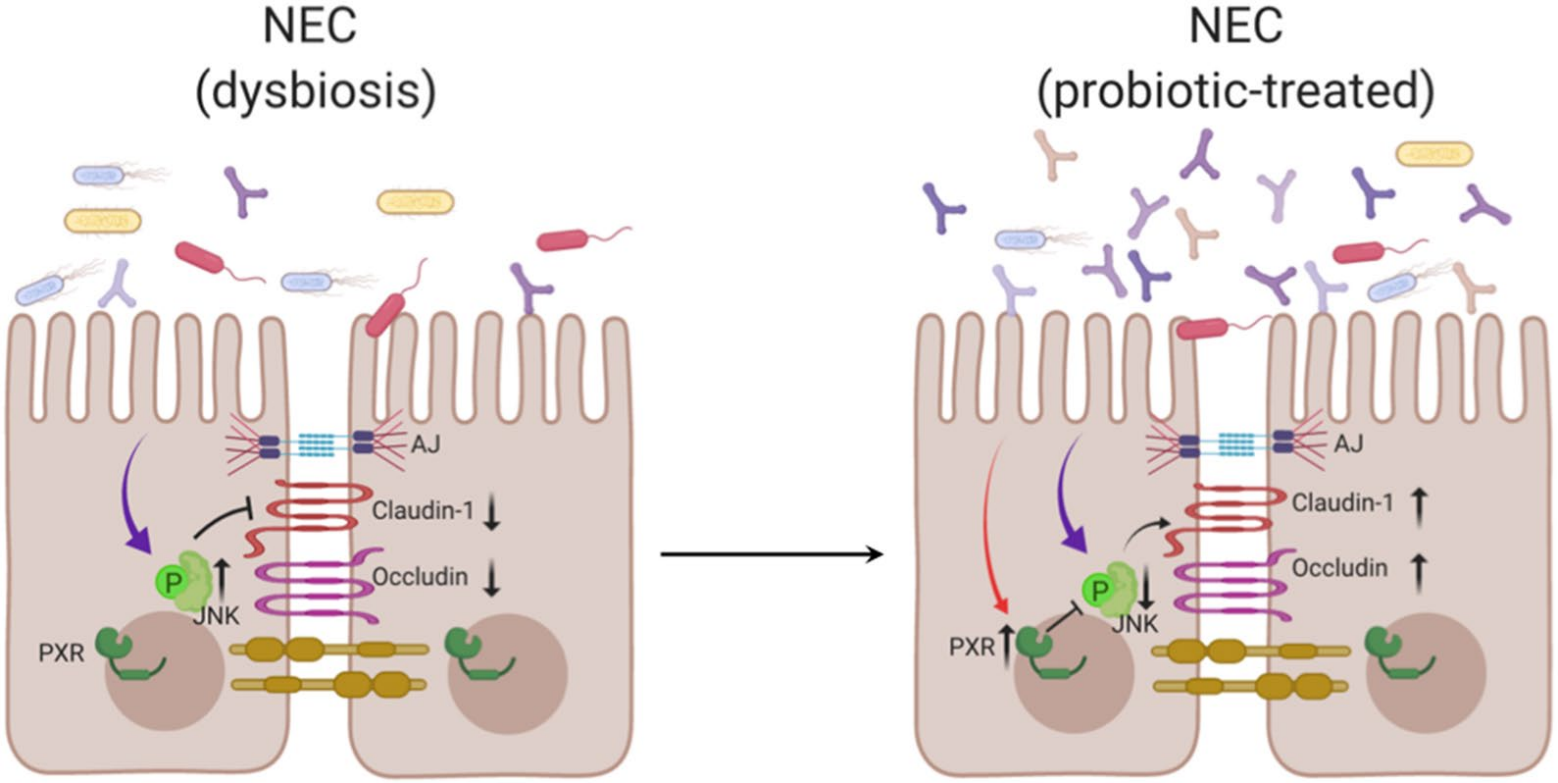
Schematic of the proposed mechanism of probiotic on enhancing intestinal epithelial barrier in NEC. The probiotics remodel the community of the intestinal microbiota, and induce the activation of PXR, which may subsequently inhibit the phosphorylation of JNK and elevate the expression of tight junction components to improve intestinal barrier function

Taken together, our results confirm the critical role of the intestinal barrier in the pathogenesis of NEC and suggest that the effectiveness of probiotics in enhancing intestinal barrier function may depend on regulation of the PXR-JNK pathway (Fig. 8). Thus, our findings help to elucidate the mechanism by which probiotics protect infants from NEC.

## Methods

### Reagents and antibodies

Lipopolysaccharide (LPS, 0111:B4) and fluorescein isothiocyanate-dextran (FD4, 60842-46-8) were purchased from Sigma-Aldrich (St. Louis, MO). The probiotic mixture is a drug recommended for children with intestinal dysfunction in the clinic and consists of *Bifidobacterium infants, Lactobacillus acidophilus, Enterococcus,* and *Bacillus cereus* (Grand Enterprises, Hangzhou, China). Primary antibodies against claudin-1 (71-7800), occludin (33-1500), and ZO-1 (61-7300) were obtained from Thermo Fisher Scientific (Waltham Mass, USA). Anti-PXR (sc-48340) was purchased from Santa Cruz Biotechnology (USA). Anti-SAPK/JNK (9252) and anti-phospho-SAPK/JNK (9255) were purchased from Cell Signaling Technology (Danvers, MA). PE-anti-phospho-JNK (562480) was purchased from BD Biosciences (Franklin Lakes, NJ).

### Experimental design and NEC model

Animal experiments were approved by the Animal Care Committee of Children's Hospital of Shanghai. All neonatal mice were randomly divided into the control group, NEC + PBS group, and NEC + probiotic group. Mice in the control group were dam-fed without hypoxia and cold stress. Necrotizing enterocolitis was induced using a modification method described by Jilling et al. [43]. Briefly, 6- to 7-day-old mice were fed 50 μl of 33% Esbilac formula (Pet-Ag) by gavage every 4 h for 96 h. Meanwhile, pups were subjected to hypoxia (99.9% N_2_ for 70 s) followed by cold stress (4 °C for 10 min) twice a day for 4 days. During the NEC modeling process, mice were fed 50 μl PBS or the probiotic mixture solution (5 × 10^4^ CFU *Bifidobacterium infants,* 5 × 10^4^ CFU *Lactobacillus acidophilus,* 5 × 10^4^ CFU *Enterococcus,* and 5 × 10^3^ CFU *Bacillus cereus*) by gavage once a day for 4 days. In all groups, the mice were weighed every morning and euthanized at 96 h. NEC was determined on histological sections of the terminal ileum according to a previously described scoring system [44]. The severity of NEC was classified as follows: intact villi were assigned a score of 0, sloughing of cells on villous tips received a score of 1, and mid-villous damage was scored as 2. An NEC score of 3 was recorded when villi were absent, but crypts were still readily detectable, and an NEC score of 4 was assigned in cases of a complete absence of epithelial structures and transmural necrosis. Scores were always determined based on the highest score observed in a specimen.

### Cell culture and transfection

The human colorectal adenocarcinoma cell line Caco-2 (from American Type Culture Collection, USA) were used to carry out in this study because of the other intestinal epithelial cell lines’ inability to form a tight monolayer [23]. Caco-2 was cultured in Dulbecco's modified Eagle's medium (DMEM) supplemented with 10% fetal bovine serum and 1% penicillin/streptomycin. Caco-2-overexpressing PXR (*Nr1i2*) cells were made by the transduction of lentiviral particles containing plasmid (pHAGE2-mCherry-*Nr1i2*). The transformants with stable integration of *Nr1i2* were obtained by a FACS Aria cell sorting system and verified by SDS-PAGE.

### Transepithelial electrical resistance (TEER)

Transwell system plates (0.4-μm pore size polyester membrane, 12-well, Costar Incorporated Corning, NY) were used for assessing intestinal barrier function in vitro. Briefly, Caco-2 cells were seeded in the apical chamber at a density of 5 × 10^5^ cells/ml and cultured for 12–14 days. The changes in TEER were measured by an epithelial volt ohmmeter (EVOM2, WPI, Sarasota, FL, USA). When the value of TEER reached at least 300 Ω·cm^2^, cells were treated with LPS.

### Paracellular permeability assay in vitro

Paracellular permeability was evaluated according to a published method [45]. Caco-2 monolayers were prepared as described above. After treatment with LPS, FD4 at a final concentration of 1 mg/ml was added to the apical layer cells and incubated at 37 °C for 12 h. By taking 100 μl from the basolateral chamber, the fluorescent signal was measured with a fluorescent plate reader using 480 nm excitation and 520 nm emission filter (SpectraMax i3).

### Gut permeability assay in vivo

The pups were orally gavaged with FD4 (80 mg/ml) for a total dose of 0.6 mg/g of body weight 4 h before killed. Whole blood was collected from the eye socket and coagulated overnight in the dark at 4 °C. Next, serum was harvested followed by centrifugation at 1000*g* for 20 min at 4 °C. Serum was diluted with PBS (pH 7.4) at 1:5, and fluorescence was measured by using a fluorescent plate reader. The serum FD4 concentrations were calculated from a standard curve obtained by serial dilution of FD4 in nontreated plasma diluted with PBS (1:5 v/v).

### Western blot analysis

Intestinal epithelial cells were isolated according to modified methods. Briefly, the small intestine was cut longitudinally and washed in PBS 3 times. Then, the tissue was transferred into a digestion solution containing 1 mM DTT, 30 mM EDTA, and 10 mM HEPES and placed in a 37 °C constant temperature shaker for 10 min. After removing residual tissue, the buffer was centrifuged at 500*g* for 5 min, and epithelial cells were collected. The whole protein of isolated epithelial cells or Caco-2 cells was extracted using the method described previously [46]. After electrophoresis in 8 or 10% polyacrylamide gel and transfer onto a PVDF membrane, the membrane was blocked for 1 h (5% BSA in TBS-Tween 20 buffer) at room temperature. Then, the membrane was incubated with primary antibody overnight at 4 °C and subsequently incubated at room temperature for 1 h with secondary antibody-conjugated HRP. Blots were developed with ECL detection reagents (Merck Millipore, USA). Relative gray was analyzed with ImageJ software.

### Quantitative real-time PCR

Total RNA extracted by using Qiagen RNA Purification kits was applied to synthesize cDNA. Real-time quantitative PCR was performed using SybrGreen (BioRad) and primers, and the reaction steps were as follows: 50 °C for 2 min, 95 °C for 10 min followed by 40 cycles at 95 °C for 10 s and then 60 °C for 30 s. All experiments were conducted in triplicate.

### Flow cytometry

Cells were stained with PE-conjugated monoclonal antibody diluted with 0.5% BSA-PBS and incubated for 30 min at 4 °C. All samples were acquired on an LSRFortessa X-20 (BD Biosciences). Data were analyzed with FlowJo 10.4 software.

### Quantitative analysis of intestinal bacteria and data analyses

The content of the small intestine was used for bacterial community analysis. After being cut longitudinally, the tissues were vigorously washed several times in PBS. The sediments from these washes were collected. All samples were immediately stored at − 80 °C. Total genomic DNA from samples was extracted using a Soil DNA Kit according to the manufacturer's protocols. After that step, indexed adapters were added to the ends of the 16S rDNA amplicons to generate indexed libraries ready for downstream NGS sequencing on Illumina sequence. After quality filtering, chimeric sequences were purified, and the resulting sequence for OTU clustering was used for VSEARCH clustering (1.9.6) (sequence similarity was set to 97%). Mean read count at a rarefied sequencing depth of 10,000 reads per sample. Then, the RDP classifier (Ribosomal Database Program) Bayesian algorithm of OTU species taxonomy analysis was used to analyze representative sequences under different species classification level statistics for the community composition of each sample. Based on OTU analysis results, using the method of random sampling, sample sequences are flat, and the Shannon index, Chao1 alpha diversity index, community species abundance and diversity of rarefaction curves can also reflect the species richness and evenness. The PCA (principal components analysis) is based on the sample OTU abundances table, PCoA (principal coordinates analysis), and NMDS (nonmetric multidimensional scaling) are based on the distance between the matrix Brary-Curtis. LEfSE analysis was used to compare the differences between the three groups.

### Statistical analysis

Statistical analysis was performed with Student’s two-tailed unpaired *t*-test and one-way or two-way analysis of variance (ANOVA) followed by Welch’s or Mann–Whitney test. For weight change analyses, two-way ANOVA was used, with treatment as the main effect. All data are expressed as mean values ± SD. *p* < 0.05 was considered to be significant. Statistical analysis was performed by GraphPad Prism 8.0.

## Supplementary Information

**Additional file 1: Fig. S1.** The subcellular localization of JNK in wild-type Caco-2 cells.

**Additional file 2: Fig. S2.** The subcellular localization of PXR in wild-type Caco-2 cells when treated with DMSO or rifaximin (1 μM) for 24 h.

## Data Availability

The datasets generated or analyzed during this study are available from the corresponding author on reasonable request.

## Abbreviations

NEC: Necrotizing enterocolitis
FD4: Fluorescein isothiocyanate-dextran 4 kDa
IEC: Intestinal epithelial cell
JNK: C-Jun N-terminal kinase
LPS: Lipopolysaccharide
MAPK: Mitogen-activated protein kinase
PXR: Pregnane X receptor
qPCR: Quantitative polymerase chain reaction
TEER: Trans-epithelial electrical resistance
TEM: Transmission electron microscope
