# Supplementary figures and images for: Probiotics mixture reinforces barrier function to ameliorate necrotizing enterocolitis by regulating PXR-JNK pathway
Source: Cell Biosci. 2021 Jan 19;11:20. doi: 10.1186/s13578-021-00530-7 (PMC7824920; doi:10.1186/s13578-021-00530-7)

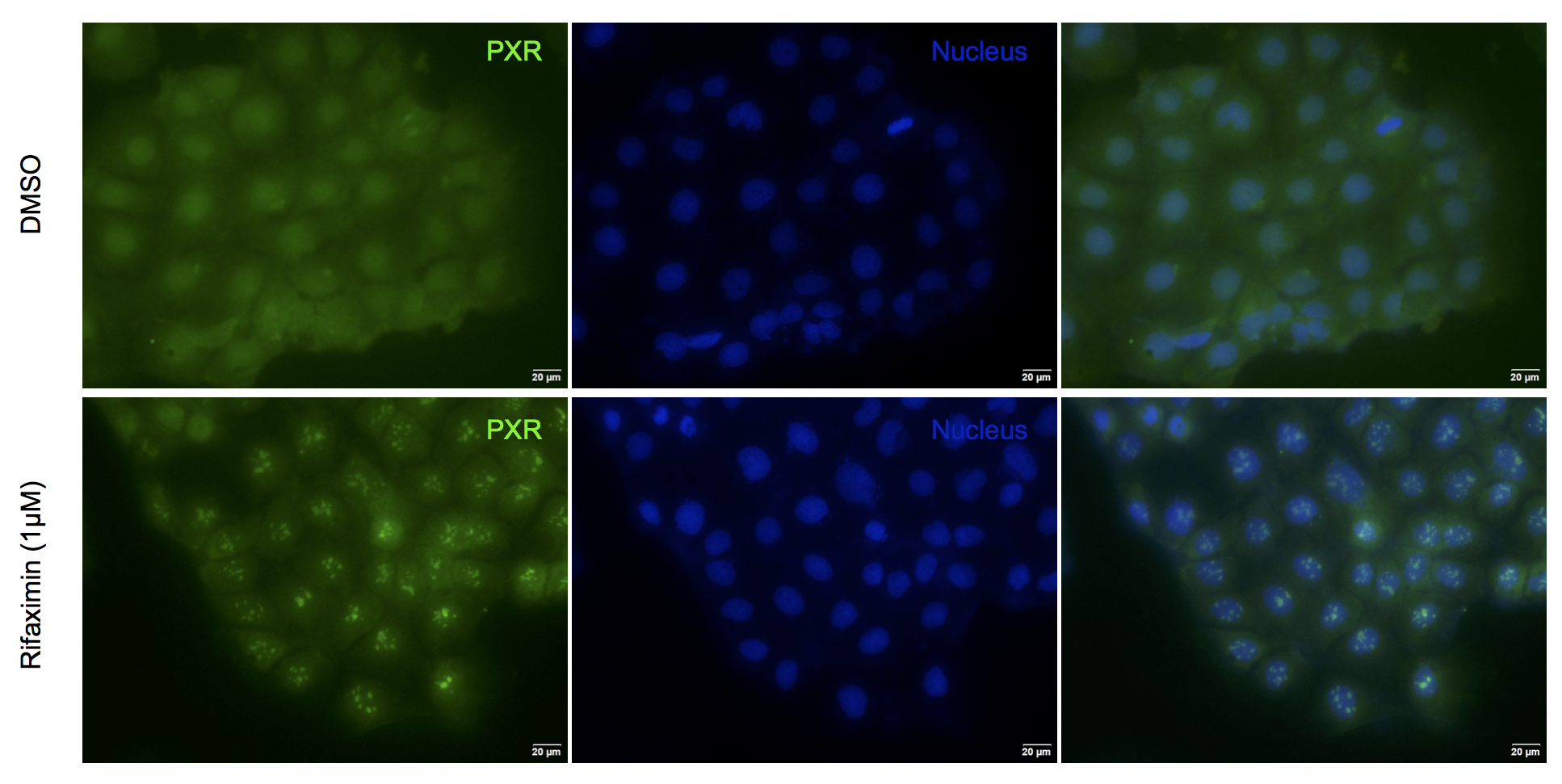

Supplement: Supplementary file 1 — Additional file 1: Fig. S1. The subcellular localization of JNK in wild-type Caco-2 cells. [file 13578_2021_530_MOESM1_ESM.png]

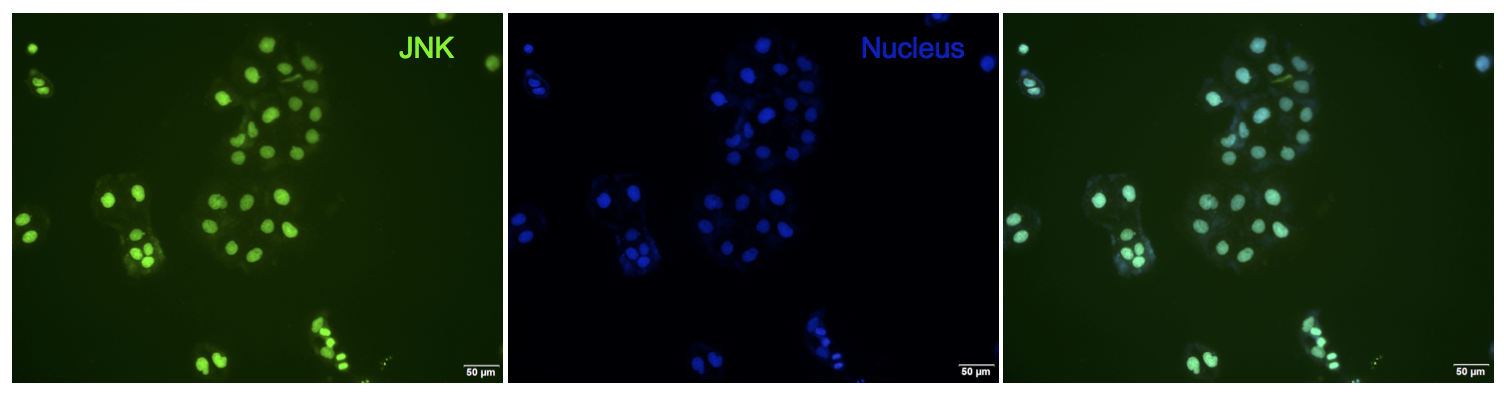

Supplement: Supplementary file 2 — Additional file 2: Fig. S2. The subcellular localization of PXR in wild-type Caco-2 cells when treated with DMSO or rifaximin (1 μM) for 24 h. [file 13578_2021_530_MOESM2_ESM.png]
